# Hemodynamic changes of donor artery after combined revascularization in adult Moyamoya disease

**DOI:** 10.1016/j.heliyon.2022.e12159

**Published:** 2022-12-09

**Authors:** Yang Dong, Lei Cao, Kaiwen Sun, Dongpeng Li, Hao Wang, Manxia Zhang, Hongwei Li, Bo Yang

**Affiliations:** Department of Neurosurgery, The First Affiliated Hospital of Zhengzhou University, Zhengzhou, China

**Keywords:** Moyamoya disease, Perioperative hemodynamic change, Color doplor ultrasonography, Combined revascularization

## Abstract

To explore the hemodynamic changes of the superficial temporal artery in adult Moyamoya Disease (MMD) who underwent combined revascularization surgery. A number of 40 patients with MMD were enrolled, and all of them underwent a direct superficial temporal artery (STA)-middle cerebral artery (STA-MCA) bypass combined with an encephalo-duro-arterio-synangiosis (EDAS). Hemodynamic parameters were detected by Color Doppler Ultrasonography (CDUS) at the preoperative, perioperative and follow-up time, including peak systolic velocity (PSV), end-diastolic velocity (EDV) and resistance index (RI). The control group were selected randomly during the same period. Researchers applied the SPSS 21 to conduct the two-sample analysis, Chi-Squared test and one-way repeated measures ANOVA between groups. *P* < 0.05 was considered statistically significant. In this study, 21 males and 19 females with an average age of 44.9 years (Range 28 y–56 y) were enrolled in the MMD group. Among them, 21 patients (52.5%) had perioperative complications, and all symptoms were transient neurological dysfunctions. Intermittent speech disorder was the most common complication during the period of operation. The preoperative hemodynamic of STA showed no significant difference between MMD and the control group. The perioperative hemodynamics had significant carnages compared with preoperative, and there was a trend of fluctuation. The perioperative PSV in the group with complications was significantly higher than the group without complications, except for EDV and RI. In the follow-up ($\bar{X}$ = 5 months), PSV (60.21 ± 22.24 cm/s, P = 0.712) showed no difference compared with baseline data, while EDV (25.12 ± 9.94 cm/s, P = 0.000) and RI (0.575 ± 0.092, P = 0.000) showed significant difference between MMD and control group. The blood flow spectrogram showed high resistance in preoperative, but most patients showed a low resistance pattern during the follow-up time. It was the first time to demonstrate that the hemodynamic changes of STA fluctuated significantly within one week and eventually remained stable after combined revascularization. The PSV may play a more important role in postoperative complications. In the follow-up, PSV had no significant difference, EDV increased significantly, and RI decreased significantly. The blood flow spectrogram mainly shows a low resistance pattern when the hemodynamic is stable.

## 1 Introduction

Moyamoya disease (MMD) is a chronic vascular disease, which is characterized by progressive stenosis and occlusion at the distal internal carotid artery, proximal portions of the anterior cerebral arteries, and middle cerebral arteries, with only a few lesions involved in the posterior circulation [1]. Though different animal models have been developed in eight species totally [2], the pathology and mechanism of MMD remains a doubt [3]. For the treatment, medical therapies and other neuro-interventional techniques [4] were proven to fail to halt the disease. More evidence indicated that direct, indirect or combined revascularization provides effective treatment in adult patients [5]. Some research teams designed the revascularization surgery to restore sufficient blood flow in cerebral hypoperfusion regions. The superficial temporal artery (STA) is the main donor vessel for surgical revascularization. Therefore, it is necessary to conduct a series of studies on the hemodynamics of extracranial and extracranial blood supply arteries. As a non-invasive, economic and reliable tool, the color doppler ultrasonography (CDUS) plays an increasingly important role in the preoperative and postoperative hemodynamic assessment of MMD [6, 7]. Some studies have stated that CDUS is an alternative tool in the evaluation of collaterals after revascularization surgery in MMD [8, 9]. Meanwhile, serial hemodynamic changes postoperatively measured by CDUS have been reported in direct revascularization [6, 7, 10] and indirect revascularization [11, 12].

However, few studies reported the hemodynamics changes which are caused by the combined revascularization, especially the dynamic of blood flow during the perioperative period. We hypothesized that perioperative complications after bypass surgery are closely related to hemodynamic fluctuations of the donor artery. In the study, perioperative hemodynamics within 10 days were monitored continuously, and the postoperative hemodynamics after three months of surgery were reported. We mainly aimed to reveal the perioperative hemodynamic changes by applying the CDUS after combined revascularization in adult MMD. To our knowledge, this is the first attempt to describe the general trend of perioperative hemodynamics within one week.

## 2 Materials and methods

### 2.1 Ethics

This study was approved by the Institutional Ethics Committee of The First Affiliated Hospital of Zhengzhou University, and written informed consent was obtained from all enrolled adult patients.

### 2.2 Patients

**Figure 1:**
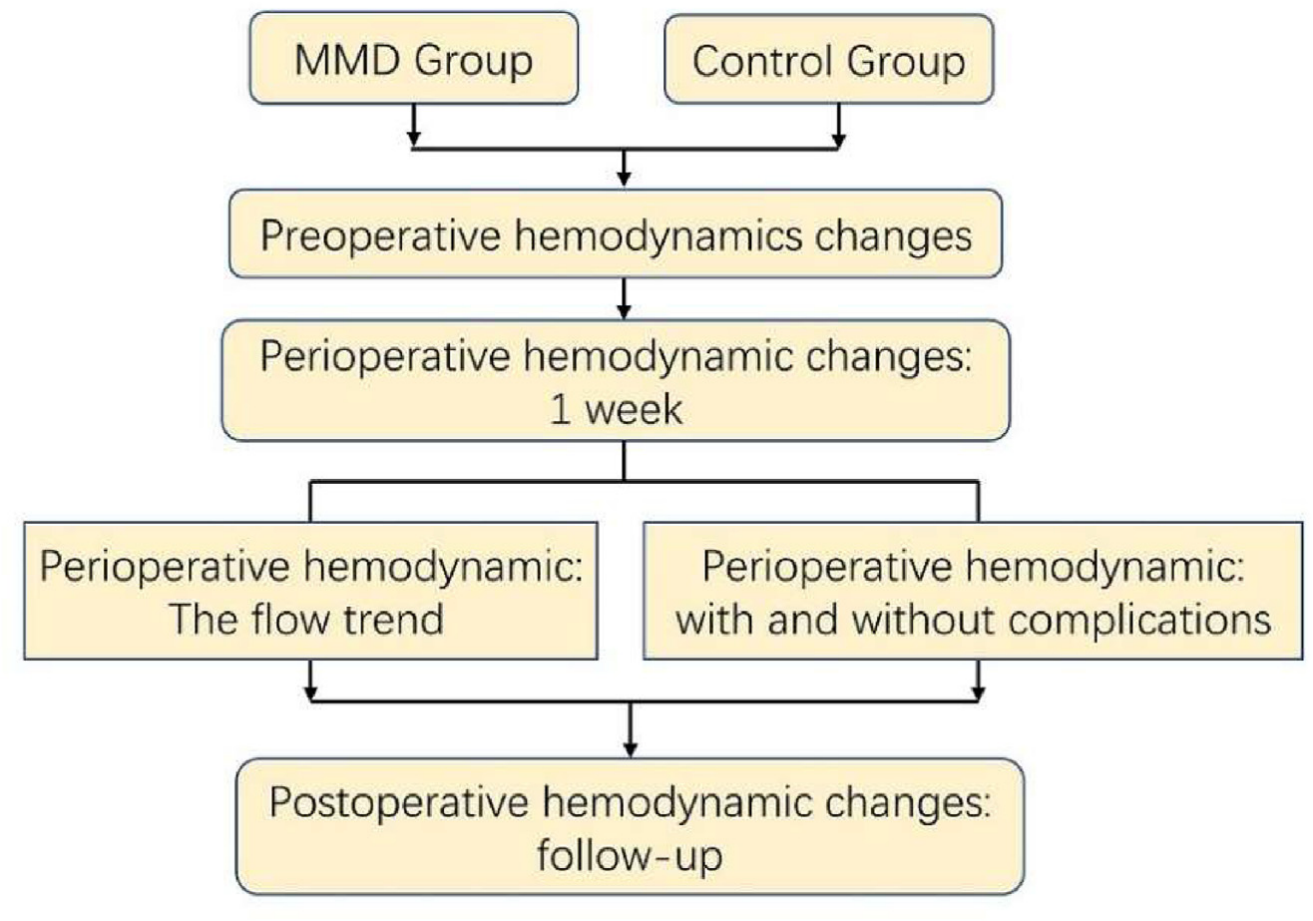
The flow chart of study design.

40 MMD patients who underwent the total procedure of combined revascularization in The First Affiliated Hospital of Zhengzhou University were enrolled in the present study. The criteria were: (a) adult patients, (b) all patients were diagnosed by digital subtraction angiography (DSA) according to the diagnostic criteria of MMD, (c) underwent the bypass surgery for the first time, (d) excluded tumor, cerebrovascular malformation and other brain diseases. Combined surgeries were carried out by the same surgeon. Intraoperative indocyanine green angiography and postoperative DSA all showed the donor artery patency. After surgery, all patients were given the same sedation management, and their blood pressure was controlled to under 140 mmHg. The study design was presented in Figure 1.

### 2.3 Surgery procedure

Combined revascularization bypass, a direct superficial temporal artery (STA) to middle cerebral artery (STA-MCA) bypass combined with an indirect encephalo-duro-arterio-synangiosis (EDAS), was conducted with standard techniques described previously [13, 14]. The STA was isolated and dissected from the scalp flap as the donor artery. Both frontal and parietal branches of STA were harvested in every case, and two branches were used. According to the symptoms of patients and DSA, the anastomosis site of MCA was selected as the recipient artery. After opening the overlying arachnoid and pia mater, an end-to-side anastomosis of the STA branches to cortical branches of MCA was performed under microscopic visualization for the direct bypass surgery. Then, for the combined bypass surgery, radial dura was inverted and inserted underneath the bone edge of the craniotomy, combined with suturing the other STA branch onto the brain surface. After then, indocyanine green was used to identify vascular patency after anastomosis. In the end, the bone flap was returned in place, the dura and scalp were then closed in layers.

### 2.4 Color Doppler ultrasonography evaluation

Hemodynamic changes were detected by the CDUS device (Tensor3300) with 3–9 MHz linear-array transducer during the preoperative period, the perioperative period within one week and three months of follow-up after surgery. CDUS examinations were performed by the same technician who was blind to the study. The hemodynamic parameters of the common STA segment were detected, including peak systolic velocity (PSV), end-diastolic velocity (EDV) and resistance index (RI), RI was calculated by the formula: RI = (PSV-EDV)/PSV. Meanwhile, 40 cases were selected randomly as the control group, and the criteria for the enrolled control group were: (a) healthy adults, (b) didn’t have a history of head surgery. The examination of the control group was performed only one time, and the same parameters were measured, including PSV, EDV and RI. All candidates were relaxed in the supine position with their head turned to the same side. The probe was placed in the trunk of the superficial temporal artery proximal to the bifurcation of the frontal and parietal branches.

### 2.5 Statistical analysis

In the analysis, categorical variables were presented as percentages and continuous variables as mean ± standard deviation. The Chi-Squared test and two-sample analysis of variance were applied to verify the difference between the surgery group and the control group. One-way repeated measures ANOVA was applied during the perioperative period, and the least-significant difference (LSD) method was used for comparison between groups. SPSS (version 21.0) was used for the analysis, and *P* < 0.05 was considered statistically significant.

## 3 Results

### 3.1 The baseline data

**Figure 2:**
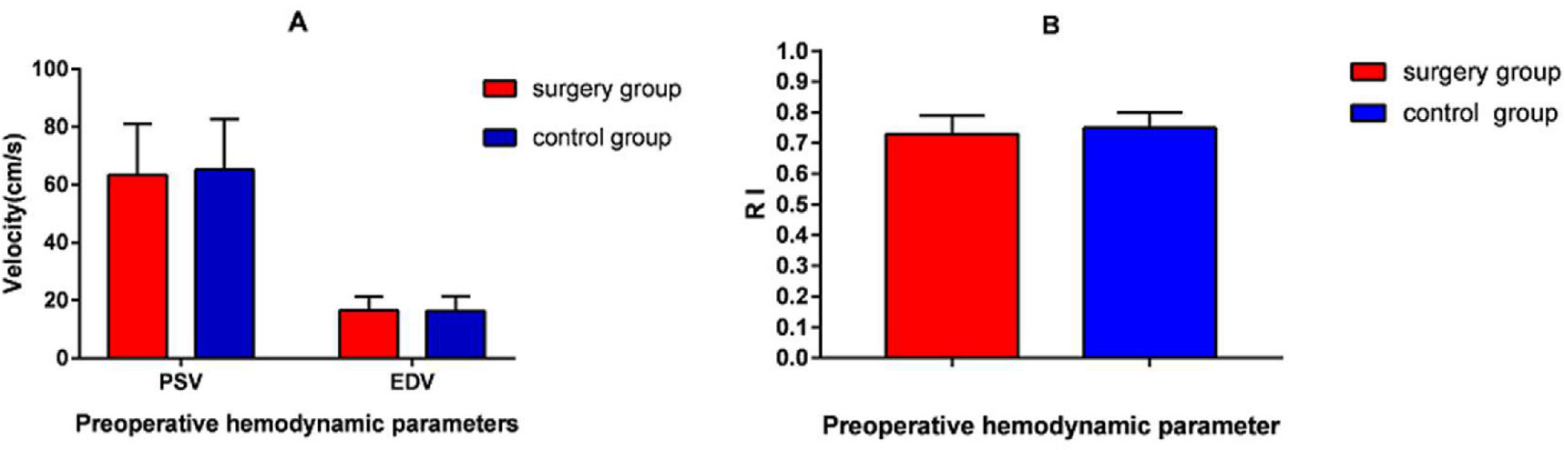
Preoperative hemodynamic changes between MMD group and control group. A. PSV and EDV of blood flow between two groups. B. RI of blood flow between two groups.

A total of 40 MMD patients (21 males and 19 females) enrolled in this study with a mean age of 44.7 years (Ranging from 28 y to 56 y). The control group consisted of 40 cases (20 males and 20 females) with a mean age of 41.8 years. For the clinical symptoms, there were four hemorrhagic cases and 36 ischemic cases. Clinical manifestations were categorized by initial symptoms: cerebral hemorrhage was the initial symptom for all of 4 hemorrhagic cases; limb dyskinesia occurred in 16 ischemic patients, main performances are limb weakness and numbness. 9 patients had aphalia. Three patients had memory decline, and the other three patients had impaired vision. Headache was also a common clinical symptom, five patients had a headache and it usually accompanied by other symptoms. 26 patients with the left side, and 14 with the right side underwent combined revascularization. The mean follow-up time was (5.4 ± 2.3) months. In the surgery group, perioperative transient neurological dysfunction after operation occurred in 21 cases (52.5%), including 18 cases of intermittent aphasia, 2 cases of Epilepsia and 1 case of sensory disturbance. All postoperative complications were transient and disappeared within one month. Compared with the control group, no significant difference was found in preoperative hemodynamic parameters of the STA in the surgery group (Figure 2A and B). The results were shown in Table 1. The baseline data of hemodynamics between MMD and control groups is shown in Table 2 and Figure 2.

**Table 1:** Clinical characteristics between MMD and control groups.

	MMD group N = 40	Control group N = 40	*P* value
Sex			*0.82*
Male	21	20	
Female	19	20	
Mean age (years)	44.9 ± 7.8	41.8 ± 9.3	*0.17*
Type			
Hemorrhagic	4	–	
Ischemic	36	–	
Side			*0.18*
Left	26	20	–
Right	14	20	
Clinical symptoms			
Headache	5		
Limb movement disorder	16		
Speech disorder	9		
Memory decline	3		
Vision decline	3		
Hematoma	4		
Follow-up time (Mean ± SD)	5.4 ± 2.3	–	–
Complications			
Intermittent aphasia	15	–	–
Seizures	2	–	–
Sensory disorder	1	–	–

**Table 2:** Preoperative hemodynamic parameters of STA between MMD and control groups.

Hemodynamic parameters (preoperative)	MMD group N = 40	Control group N = 40	*P* value
PSV	63.41 ± 17.61	65.25 ± 17.44	0.66
EDV	16.60 ± 4.77	16.37 ± 5.06	0.85
RI	0.73 ± 0.06	0.75 ± 0.05	0.20

### 3.2 Perioperative hemodynamic changes within 10 days

**Figure 3:**
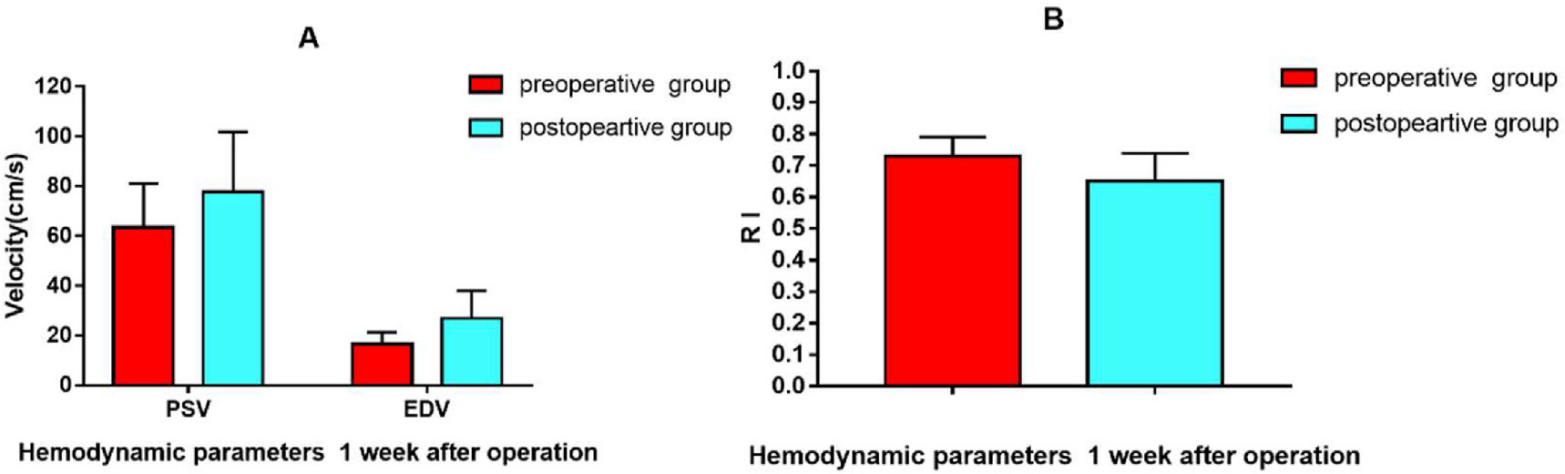
Hemodynamic changes 1 week after operation. A. PSV and EDV of blood flow between preoperative group and 1 week postoperative group B. RI of blood flow between two groups.

First, as some studies demonstrated the hemodynamic changes one week or two weeks after the operation, we elevated the postoperative hemodynamic parameters of STA after one week. Compared with the preoperative, the PSV and EDV revealed a significant increase (Figure 3A), while RI revealed a significant decrease one week after the operation (Figure 3B). Hemodynamic results one week after the operation are shown in Table 3 and Figure 3.

**Table 3:** Hemodynamic changes of STA 1 week after operation.

Hemodynamic parameters	Preoperative group N = 40	Perioperative group (1 week) N = 40	*P* value
PSV	63.41 ± 17.61	77.62 ± 24.21	0.003
EDV	16.60 ± 4.77	26.72 ± 11.35	0.000
RI	0.73 ± 0.06	0.65 ± 0.09	0.001

**Figure 4:**
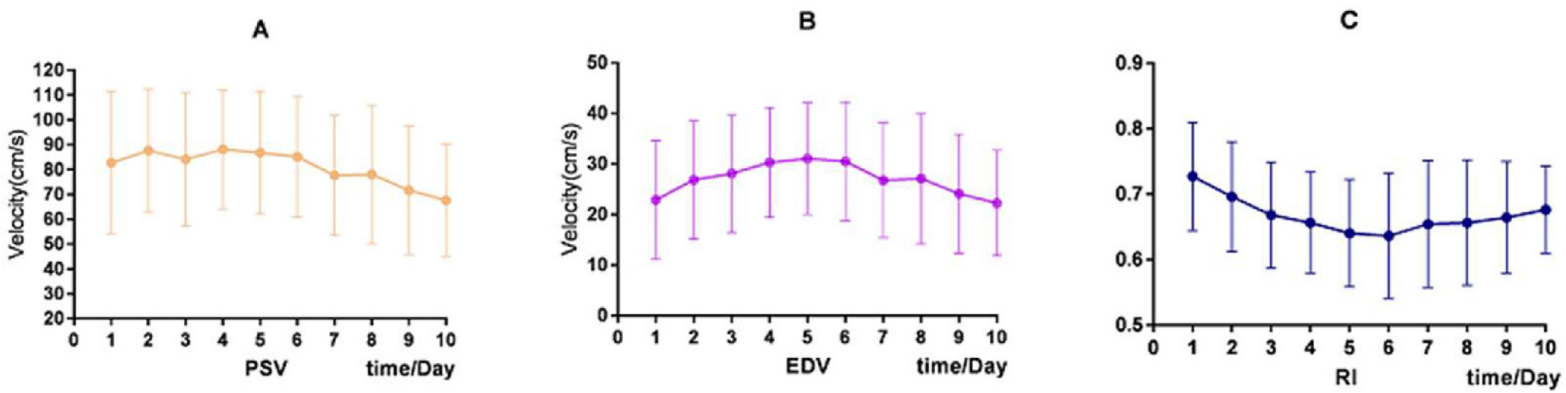
Perioperative hemodynamic changes of STA within 10 days. A hemodynamic change of PSV. B hemodynamic change of EDV; C hemodynamic change of RI.

Next, the postoperative hemodynamic parameters per day were compared to the preoperative data. The perioperative hemodynamic changes of STA within ten days increased significantly compared with preoperative (P < 0.05), while RI decreased, but there was no significant difference. From the second day to the seventh day, PSV, EDV and RI all had significant changes compared with preoperative. Overall, the PSV revealed an alternate change of rising and falling (Figure 4A), EDV increased first and reached a peak five days after the operation (Figure 4B), RI decreased until reaching the lowest level on the 6th day postoperatively (Figure 4C). PSV within eight days and EDV within ten days after operation changed significantly compared with the previous day. RI within ten days after the operation changed considerably except for the first day. The decreased RI mainly depended on the rise of EDV. The trend of the perioperative hemodynamic changes in the STA was illustrated in Table 4 and Figure 4.

**Table 4:** Perioperative hemodynamic changes of STA compared with preoperative.

Time	PSV	*P*	EDV	*P*	RI	*P*
1st	82.73 ± 28.65	0.001	22.87 ± 11.74	0.012	0.727 ± 0.083	0.846
2nd	87.64 ± 24.80	0.000	26.82 ± 11.68	0.000	0.696 ± 0.084	0.035
3rd	84.12 ± 26.90	0.001	28.07 ± 11.62	0.000	0.668 ± 0.081	0.002
4th	88.11 ± 24.02	0.000	30.29 ± 10.88	0.000	0.656 ± 0.078	0.000
5th	86.78 ± 24.77	0.000	31.03 ± 11.06	0.000	0.640 ± 0.082	0.000
6th	85.09 ± 24.35	0.000	30.47 ± 11.77	0.000	0.636 ± 0.096	0.000
7th	77.62 ± 24.21	0.003	26.72 ± 11.35	0.000	0.654 ± 0.097	0.001
8th	78.03 ± 27.80	0.023	27.07 ± 12.86	0.000	0.656 ± 0.096	0.000
9th	71.65 ± 25.76	0.122	24.08 ± 11.77	0.000	0.664 ± 0.086	0.000
10th	67.65 ± 22.58	0.501	22.24 ± 10.41	0.002	0.676 ± 0.067	0.001

**Figure 5:**
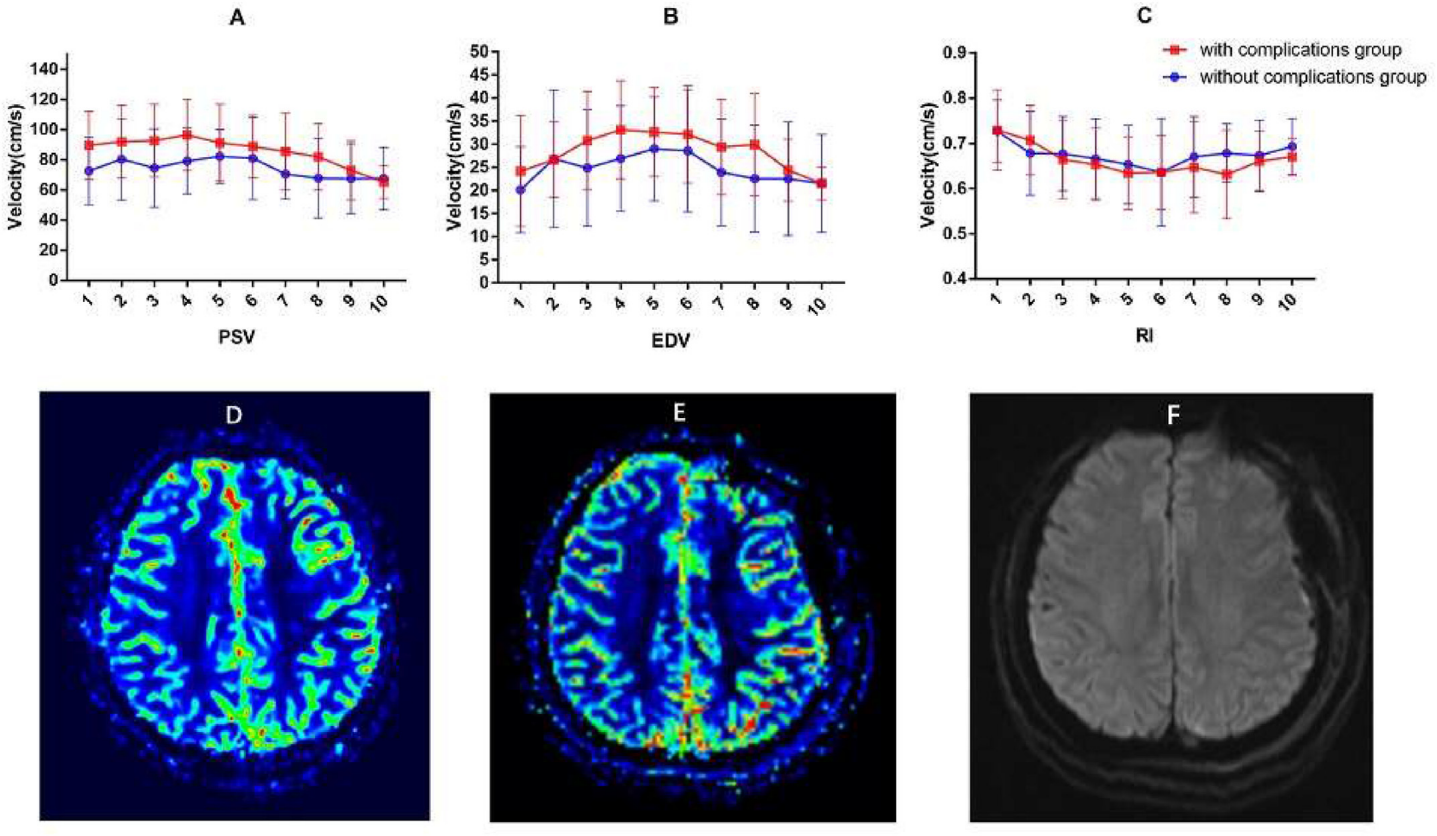
Perioperative hemodynamic changes of STA between without complication group and with complication group. A hemodynamic change of PSV. B hemodynamic change of EDV; C hemodynamic change of RI; D image of preoperative CBF; E image of perioperative CBF; F image of perioperative DWI. Warm color indicates high blood flow, cold color indicates low blood flow.

Finally, the perioperative hemodynamic data were divided into two groups according to whether patients had complications or not. The diverse CDUS parameters between the two groups were further analyzed. For PSV, a significant difference was found on the first day, third day and 4th day postoperatively. However, the EDV and RI were not found the significant difference between the two groups, PSV and EDV of patients with complications seemed higher than the group without complications. The results are presented with Table 5 and Figure 5(A, B, C). The cerebral blood flow (CBF) data of a female patients with the age of 52 y were illustrated in Figure 5(D, E, F). Intermittent aphasia was detected during the perioperative period. Perioperative perfusion weighted imaging showed an elevated CBF. Cerebral infarction was not detected in diffusion weighted imaging (DWI). Encephaledema was also not detected in DWI, which is usually presented in HS.

**Table 5:** Perioperative hemodynamic changes of STA between without complication group and with complication group.

Time	PSV			EDV			RI		
	Without group	With group	*P*	Without group	With group	*P*	Without group	With group	*P*
1st	72.70 ± 22.38	89.66 ± 22.54	0.040	20.15 ± 9.31	24.16 ± 11.98	0.305	0.727 ± 0.069	0.729 ± 0.089	0.945
2nd	80.44 ± 27.01	92.07 ± 18.80	0.154	26.85 ± 14.82	26.65 ± 8.12	0.960	0.678 ± 0.093	0.707 ± 0.077	0.339
3rd	74.52 ± 25.98	92.76 ± 24.11	0.046	24.84 ± 12.56	30.81 ± 10.60	0.149	0.677 ± 0.082	0.664 ± 0.086	0.674
4th	79.19 ± 21.78	96.54 ± 23.74	0.040	26.92 ± 11.39	33.15 ± 10.62	0.116	0.666 ± 0.088	0.653 ± 0.080	0.660
5th	82.25 ± 17.77	91.36 ± 25.47	0.258	29.00 ± 11.29	32.66 ± 9.67	0.326	0.653 ± 0.087	0.634 ± 0.081	0.515
6th	81.02 ± 27.34	88.93 ± 20.77	0.352	28.62 ± 13.21	32.14 ± 10.53	0.400	0.636 ± 0.119	0.636 ± 0.082	0.986
7th	70.59 ± 16.49	85.62 ± 25.29	0.062	23.95 ± 11.59	29.40 ± 10.34	0.165	0.670 ± 0.089	0.647 ± 0.101	0.501
8th	67.80 ± 26.41	82.09 ± 21.97	0.100	22.58 ± 11.61	29.93 ± 11.06	0.074	0.678 ± 0.065	0.631 ± 0.098	0.129
9th	67.56 ± 23.10	73.11 ± 19.52	0.460	22.50 ± 12.27	24.40 ± 6.72	0.572	0.673 ± 0.078	0.660 ± 0.067	0.592
10th	67.65 ± 20.77	65.26 ± 10.87	0.672	21.51 ± 10.59	21.50 ± 3.51	0.997	0.693 ± 0.062	0.670 ± 0.041	0.196

### 3.3 Postoperative hemodynamic changes in follow-up

**Figure 6:**
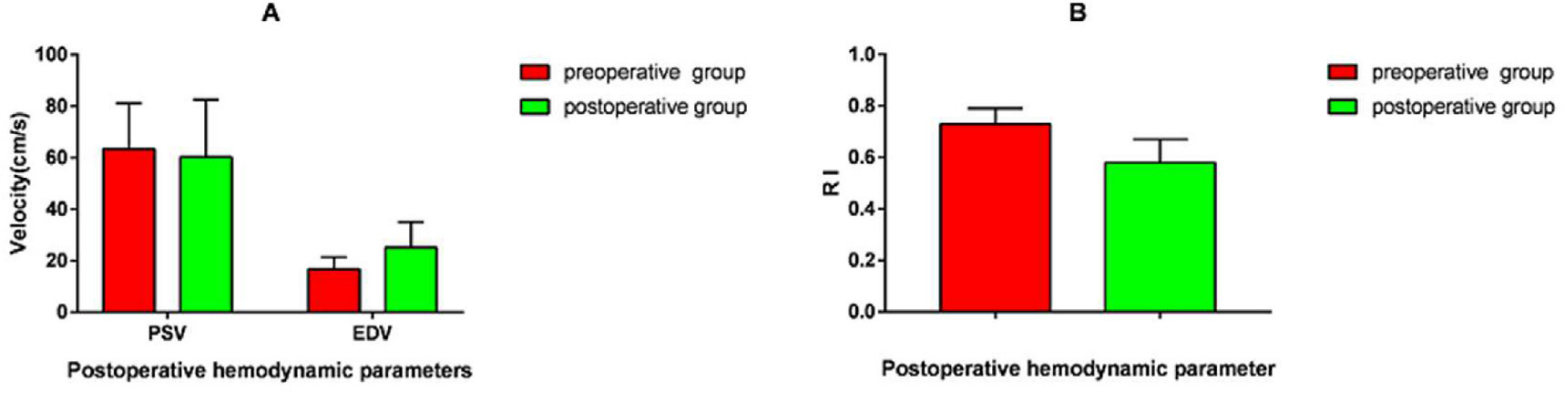
Postoperative hemodynamic changes of STA in follow-up between the preoperative group and the postoperative group. A. PSV and EDV of blood flow between two groups. B. resistance index of blood flow between two groups.

**Figure 7:**
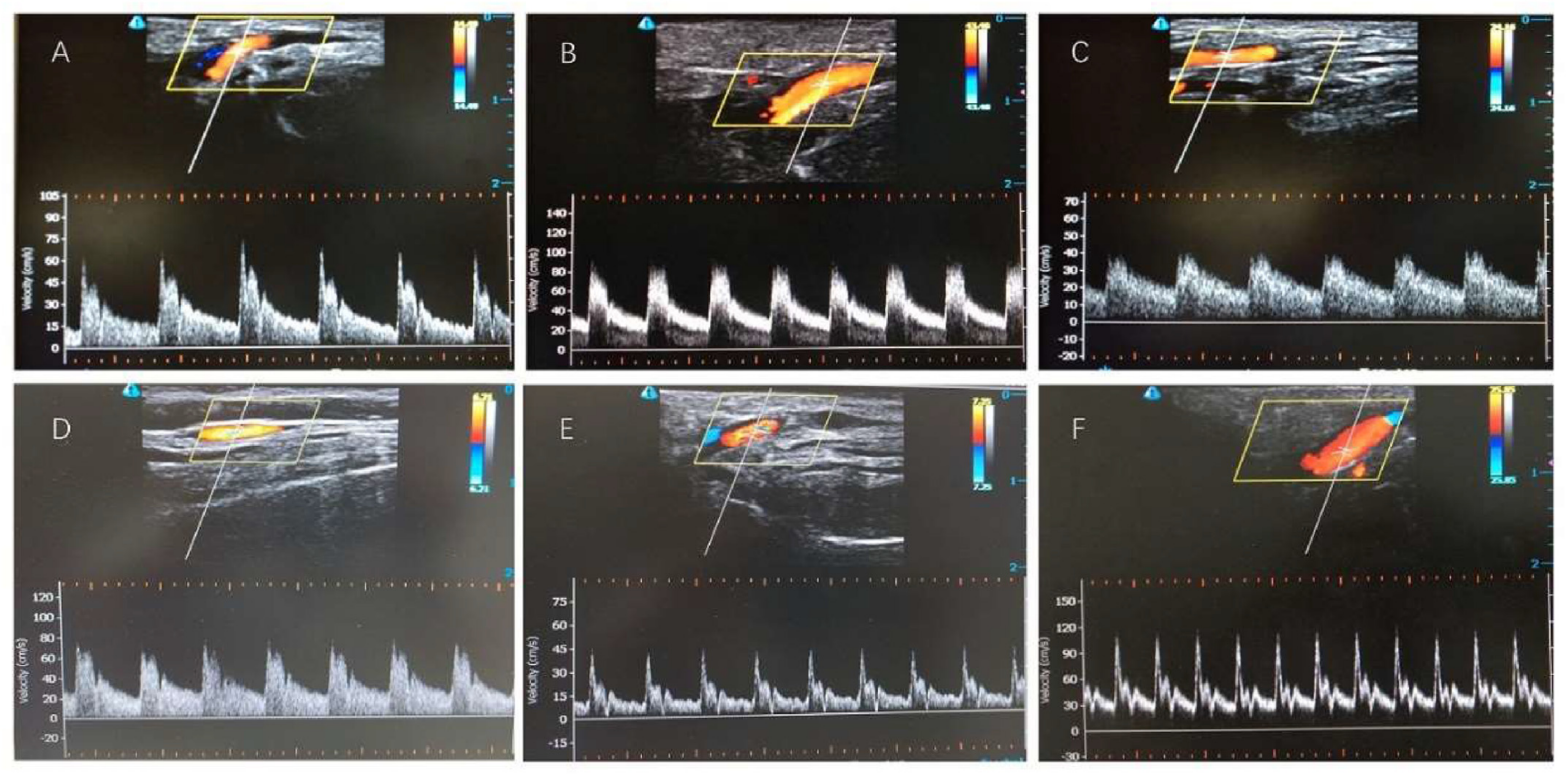
Color Doppler spectrogram of the STA blood flow between low and high resistant pattern. A. preoperative spectrogram of the low resistant pattern B. perioperative spectrogram of the low resistant pattern C. postoperative spectrogram in the follow-up of the low resistant pattern D. preoperative spectrogram of the high resistant pattern E. perioperative spectrogram of the high resistant pattern F. postoperative spectrogram in the follow-up of the high resistant pattern.

After the operation, PSV, EDV and RI were significantly reduced in the follow-up of five months. For the examination of CDUS, the spectrogram of the STA showed a high resistance pattern with a spike shape before the operation. As time went on, the feature of spike shape in the spectrogram gradually disappeared and was eventually replaced by a gentle peak shape. Among most patients, the peak was predominantly presented with a platform but not a point. Research results are shown in Table 6 and Figure 6(A, B). The spectrogram of CDUS is illustrated in Figure 7(A, B, C).

**Table 6:** Postoperative hemodynamic changes of STA in follow-up.

	Preoperative group N = 40	Postoperative group N = 40	*P* value
PSV	63.41 ± 17.61	60.21 ± 22.24	0.80
EDV	16.60 ± 4.77	25.12 ± 9.94	0.00
RI	0.73 ± 0.06	0.58 ± 0.09	0.00

**Figure 8:**
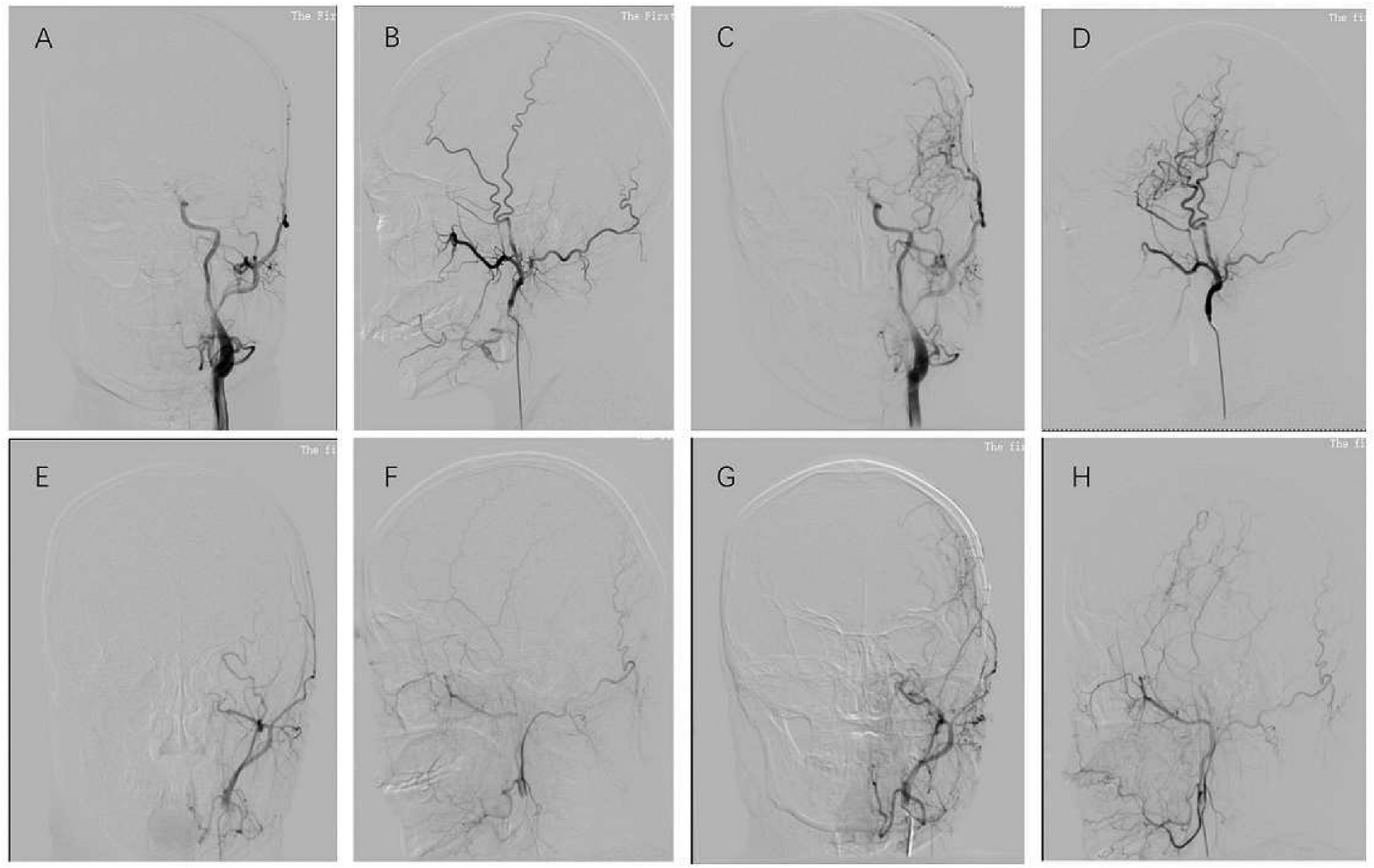
The angiogram of collateral development between low resistant pattern and high resistant pattern. A. preoperative coronal DSA of low resistant pattern, B. preoperative sagittal DSA of low resistant pattern, C. postoperative coronal DSA of low resistant pattern, D. postoperative sagittal DSA of low resistant pattern, E. preoperative coronal DSA of high resistant pattern, F. preoperative sagittal DSA of high resistant pattern, G. postoperative coronal DSA of high resistant pattern, H. postoperative sagittal DSA of high resistant pattern.

However, the spectrogram with a sharply decreased peak seems to change little in some patients. For these patients, the postoperative characteristic of the Color Doppler spectrum was similar to preoperative with a sharp systolic peak. Compared with most gentle peak shapes in the perioperative spectrogram pattern, the spectrogram pattern of these patients showed a different feature of spike shape. In other words, the spectrogram pattern of minor patients changed little among preoperative, perioperative and postoperative. The spectrogram of CDUS is illustrated in Figure 7(D, E, F). The CDUS spectrogram showed a low resistant pattern in most of the patients but a high pattern in a few patients; differences in the collateral development between the two groups existed. Compared with the low resistant pattern group (Figure 8A, B, C, D), the collateral development of the high resistant pattern group was poorer (Figure 8E, F, G, H). The preoperative and postoperative angiogram of the two patterns are presented in Figure 8.

## 4 Discussion

The intracranial blood flow has complex hemodynamic changes after combined revascularization in adult MMD and then influences the collateral network indirectly after revascularization procedures [15]. Many imaging techniques are available to evaluate the donor vessel after bypass surgery [16]. Some studies have addressed the hemodynamic changes in STA by CDUS. Wang et al [17] reported that no differences were found in PSV, EDV, and RI in baseline, between two weeks and six months after the operation, except for the significant increase in PSV and EDV between preoperative and two weeks after the operation. In our study, the significant increase in PSV and EDV and the significant decrease in RI were also found one week after operation compared with preoperative. The hemodynamic changes of STA may be related to the extracranial vessels, which belong to the high resistance system, while the intracranial vessels belong to the low resistance system. Meanwhile, the decreased RI indicated a low resistance in the cerebral perfusion. However, the mechanism in STA resistance reduction quickly after combined revascularization is still unclear, and further exploration is required. Significantly, we firstly evaluated the continuous hemodynamic changes in the perioperative period after combined revascularization and observed a fluctuating trend of perioperative hemodynamic changes. We found the result that the hemodynamics remained in an extremely unstable state during the perioperative period, especially one week after the operation. To further explore the perioperative hemodynamics, patients were divided into two groups and further analyzed. One group is those patients who had postoperative complications, and the other group is those who are without complications. The Significant difference was detected in perioperative PSV between the two groups, but the situation of EDV and RI is the opposite. The PSV and EDV presented a relatively higher level in the group with perioperative complications. Therefore, the results indicated that PSV might play a more important role in perioperative complications. Given those interesting findings, potential bias also existed in the research. One was the small sample between two groups, the other was that postoperative complications occurred at different times. These factors may have an influence on the accuracy of the data.

Vascular reconstruction could result in heterogeneous hemodynamic, CBF at the site of the anastomosis grew fast after revascularization, and particular surgical complications appeared [18, 19], which could be categorized as neurological and non-neurological complications [20]. In our study, a high incidence of perioperative complications was observed, and over fifty percent of the patients presented non-neurological complications. All symptoms were temporary and disappeared within one month. Meanwhile, there were no permanent complications among the patients. Transient speech disorder was the most common perioperative disorder; one of the main reasons may be the large operation volume on the left side. And the unstable hemodynamics also probably has a close relationship with this cause. It’s reported that hyperperfusion syndrome (HS) and postoperative cerebral hemorrhage/infarction were the most common surgical complications related to hemodynamics, with a maximum incidence of 50% [21, 22]. In most cases, HS usually occurred within the first week and improved within the second week after operation [23, 24, 25, 26]. According to previous studies, cerebral perfusion increased significantly higher after direct revascularization [27, 28, 29]. However, an interesting fact is found that HS did not occur in all patients. An equally important finding is that MMD patients demonstrated a higher HS incidence than atherosclerotic carotid artery occlusion patients, which possibly resulted from vascular reconstruction or great hemodynamic changes after bypass surgery [15, 20, 25, 30]. Furthermore, some studies have also pointed out that hemodynamic fluctuations are probably the main cause of MMD complications in the perioperative period [31]. Given our observation, that fluctuation of the perioperative hemodynamic changes is the main feature of STA after combined revascularization, which was consistent with the above conclusions. As most of the research implied a close relationship between HS and hemodynamic change, we hypothesized that perioperative complications might be mostly related to the unstable intracranial collateral hemodynamic and the redistribution of intracranial blood flow. Therefore, the present study directly showed the perioperative hemodynamic characteristics and provided a new sight for the prevention of HS. It’s indicated that optimal perioperative hemodynamic management would be proved to avoid these deleterious phenomena after surgery.

In follow-up, we conducted an analysis of the hemodynamic changes in our study. The results indicated that a significant increase in EDV and a significant decrease in RI between preoperative and five months follow-up, but there were no significant changes in PSV during this period. It was indicated that the decreased RI was mainly depended on the significantly increase in EDV. The main reason why our research is different from Wang et al [17] is that different indirect revascularization which Wang combined with EDMS, while the present study combined with EDAS. Considering the continuous changes of hemodynamic parameters in different periods, we hypothesized that a fluctuating trend exists in hemodynamic changes of STA after combined revascularization and achieves an appropriate level ultimately. PSV will have a significant fluctuation and eventually return to the original preoperative level, EDV will have a significant increase and eventually stabilize at a higher hemodynamic velocity than baseline, and RI will have a significant increase and eventually remain stable. Additionally, the feature of the blood flow spectrogram in the CDUS was surveyed in different periods, which is characterized by a high resistance pattern with a peak shape in the preoperative and the low resistance pattern formed gradually in the perioperative period. In the follow-up time, the spectrogram mainly showed a low residence pattern without the obvious systolic peak. The spectrogram indicated a low resistance of the intracranial vessels and the establishment of collaterals. However, a few patients still presented a high resistance pattern. The most likely reason is the differences in the growth speed of neovascularization and the development of collateral branches among patients.

Certainly, there are still some potential limitations in our study. First, we only evaluated three hemodynamic parameters of the STA. If combined with mean flow velocity, mean velocity ratio, mean flow rate ratios and PI, these parameters may obtain more information. Second, frontal and parietal branches of the donor surgical artery were chosen as sample due to the hypoperfusion region and differed from each patient. It is possible that the degree of hypoperfusion and branch of the donor artery may affect the hemodynamics. Third, the sample was small when conducting the analysis between groups which are with complications and groups which are without complications.

In conclusion, the hemodynamic changes that STA had significant fluctuation within one week and eventually remained stable after combined revascularization. Compared with the preoperative, PSV and EDV increased significantly, whereas RI decreased significantly during the perioperative period. The PSV may play a more important role in postoperative complications. In the follow-up, PSV showed no significant difference, EDV increased significantly, and RI decreased significantly. The pictures of blood streams mainly showed the low resistance pattern when hemodynamic is stable.

## Declarations

### Author contribution statement

Yang Dong, Lei Cao: Performed the experiments; Wrote the paper.

Kaiwen Sun, Dongpeng Li, Hao Wang: Analyzed and interpreted the data.

Manxia Zhang: Contributed reagents, materials, analysis tools or data.

Hongwei Li; Bo Yang: Conceived and designed the experiments.

### Funding statement

This research did not receive any specific grant from funding agencies in the public, commercial, or not-for-profit sectors.

### Data availability statement

Data included in article/supp. material/referenced in article.

### Declaration of interest’s statement

The authors declare no conflict of interest.

### Additional information

No additional information is available for this paper.

## References

[1] Deng X. (2017). Moyamoya disease with occlusion of bilateral vertebral arteries and the basilar artery fed by the collateral vessels of vertebral arteries: a rare case report. J. Clin. Neurosci..

[2] Montaser A.S. (2022). Ivy sign: a diagnostic and prognostic biomarker for pediatric moyamoya. J. Neurosurg. Pediatr..

[3] Berry J.A. (2020). Moyamoya: an update and review. Cureus.

[4] Khan N. (2011). Failure of primary percutaneous angioplasty and stenting in the prevention of ischemia in Moyamoya angiopathy. Cerebrovasc. Dis..

[5] Funaki T. (2014). Incidence of late cerebrovascular events after direct bypass among children with moyamoya disease: a descriptive longitudinal study at a single center. Acta Neurochir. (Wien).

[6] Jin S.W. (2017). Increased ratio of superficial temporal artery flow rate after superficial temporal artery-to-middle cerebral artery anastomosis: can it reflect the extent of collateral flow?. World Neurosurg..

[7] Kraemer M. (2012). Postoperative changes in the superficial temporal artery and the external carotid artery duplex sonography after extra-intracranial bypass surgery in European Moyamoya disease. Clin. Neurol. Neurosurg..

[8] Pan H.W. (2016). Color Doppler ultrasonography in the evaluation of compensatory arteries in patients with moyamoya disease: combined with cerebral angiography. Eur. Rev. Med. Pharmacol. Sci..

[9] Yeh S.J. (2017). Color Doppler ultrasonography as an alternative tool for postoperative evaluation of collaterals after indirect revascularization surgery in Moyamoya disease. PLoS One.

[10] Wu M. (2011). Color Doppler hemodynamic study of the superficial temporal arteries in superficial temporal artery-middle cerebral artery (STA-MCA) bypass surgery for Moyamoya disease. World Neurosurg..

[11] Yeh S.J. (2017). Color Doppler ultrasonography as an alternative tool for postoperative evaluation of collaterals after indirect revascularization surgery in Moyamoya disease. PLoS One.

[12] Ogawa S. (2017). Acceleration of blood flow as an indicator of improved hemodynamics after indirect bypass surgery in Moyamoya disease. Clin. Neurol. Neurosurg..

[13] Zhao Y. (2018). Direct bypass surgery vs. Combined bypass surgery for hemorrhagic moyamoya disease: a comparison of angiographic outcomes. Front. Neurol..

[14] Zhang M. (2020). Combined STA-MCA bypass and encephalodurosynangiosis versus encephalodurosynangiosis alone in adult hemorrhagic moyamoya disease: a 5 -year outcome study. J. Stroke Cerebrovasc. Dis..

[15] Fujimoto S. (2013). Changes in superficial temporal artery blood flow and cerebral hemodynamics after extracranial-intracranial bypass surgery in moyamoya disease and atherothrombotic carotid occlusion. J. Neurol. Sci..

[16] Ashley W.W. (2008). Flow-assisted surgical cerebral revascularization. Neurosurg. Focus.

[17] Wang Y. (2014). Hemodynamic study with duplex ultrasonography on combined (direct/indirect) revascularization in adult moyamoya disease. J. Stroke Cerebrovasc. Dis..

[18] Ogasawara K. (2005). Neural damage caused by cerebral hyperperfusion after arterial bypass surgery in a patient with moyamoya disease: case report. Neurosurgery.

[19] Fujimura M. (2009). Delayed intracerebral hemorrhage after superficial temporal artery-middle cerebral artery anastomosis in a patient with moyamoya disease: possible involvement of cerebral hyperperfusion and increased vascular permeability. Surg. Neurol..

[20] Fujimura M., Tominaga T. (2015). Significance of cerebral blood flow analysis in the acute stage after revascularization surgery for moyamoya disease. Neurol. Med.-Chir.(Tokyo).

[21] Ahn I.M. (2014). Incidence, prevalence, and survival of moyamoya disease in Korea: a nationwide, population-based study. Stroke.

[22] Yu J. (2016). Progress on complications of direct bypass for moyamoya disease. Int. J. Med. Sci..

[23] Fujimura M. (2007). Temporary neurologic deterioration due to cerebral hyperperfusion after superficial temporal artery-middle cerebral artery anastomosis in patients with adult-onset moyamoya disease. Surg. Neurol..

[24] Fujimura M. (2009). Incidence and risk factors for symptomatic cerebral hyperperfusion after superficial temporal artery-middle cerebral artery anastomosis in patients with moyamoya disease. Surg. Neurol..

[25] Fujimura M. (2011). Significance of focal cerebral hyperperfusion as a cause of transient neurologic deterioration after extracranial-intracranial bypass for moyamoya disease: comparative study with non-moyamoya patients using N-isopropyl-p-[(123)I]iodoamphetamine single-photon emission computed tomography. Neurosurgery.

[26] Ohue S. (2008). Postoperative temporary neurological deficits in adults with moyamoya disease. Surg. Neurol..

[27] Lee M. (2011). Intraoperative blood flow analysis of direct revascularization procedures in patients with moyamoya disease. J. Cerebr. Blood Flow Metabol..

[28] Badie B. (2000). Intraoperative sonographic assessment of graft patency during extracranial-intracranial bypass. AJNR Am. J. Neuroradiol..

[29] Cheung A.H. (2017). Surgical outcome for moyamoya disease: clinical and perfusion computed tomography correlation. World Neurosurg..

[30] Hayashi K. (2012). Incidence and clinical features of symptomatic cerebral hyperperfusion syndrome after vascular reconstruction. World Neurosurg..

[31] Funaki T. (2015). Unstable moyamoya disease: clinical features and impact on perioperative ischemic complications. J. Neurosurg..

